# Dose‐dependent effects of esketamine on brain activity in awake mice: A BOLD phMRI study

**DOI:** 10.1002/prp2.1035

**Published:** 2022-12-12

**Authors:** Kyrsten Kawazoe, Ryan McGlynn, Wilder Felix, Raquel Sevilla, Siyang Liao, Praveen Kulkarni, Craig F. Ferris

**Affiliations:** ^1^ Department of Pharmaceutical Sciences Northeastern University Boston Massachusetts USA; ^2^ Center for Translational Neuroimaging Northeastern University Massachusetts Boston USA; ^3^ Department of Psychology Northeastern University Boston Massachusetts USA

**Keywords:** depression, hippocampus, NMDA, phMRI, prefrontal cortex

## Abstract

Pharmacological magnetic resonance imaging (phMRI) is a noninvasive method used to evaluate neural circuitry involved in the behavioral effects of drugs like ketamine, independent of their specific biochemical mechanism. The study was designed to evaluate the immediate effect of esketamine, the S‐isomer of (±) ketamine on brain activity in awake mice using blood oxygenation level dependent (BOLD) imaging. It was hypothesized the prefrontal cortex, hippocampus, and brain areas associated with reward and motivation would show a dose‐dependent increase in brain activity. Mice were given vehicle, 1.0, 3.3, or 10 mg/kg esketamine I.P. and imaged for 10 min post‐treatment. Data for each treatment were registered to a 3D MRI mouse brain atlas providing site‐specific information on 134 different brain areas. There was a global change in brain activity for both positive and negative BOLD signal affecting over 50 brain areas. Many areas showed a dose‐dependent decrease in positive BOLD signal, for example, cortex, hippocampus, and thalamus. The most common profile when comparing the three doses was a U‐shape with the 3.3 dose having the lowest change in signal. At 1.0 mg/kg there was a significant increase in positive BOLD in forebrain areas and hippocampus. The anticipated dose‐dependent increase in BOLD was not realized; instead, the lowest dose of 1.0 mg/kg had the greatest effect on brain activity. The prefrontal cortex and hippocampus were significantly activated corroborating previous imaging studies in humans and animals. The unexpected sensitivity to the 1.0 mg/kg dose of esketamine could be explained by imaging in fully awake mice without the confound of anesthesia and/or its greater affinity for the N‐methyl‐d‐aspartate receptor (NMDAR) receptor than (±) ketamine.

## Graphical abstract

Low dose esketamine activates prefrontal cortex. One milligram per Kilogram of esketamine given during the MRI scanning session to awake mice activates areas in the prefrontal cortex while higher doses are less effective. This dose dependent pattern of BOLD activation included the hippocampus but not reward circuitry.

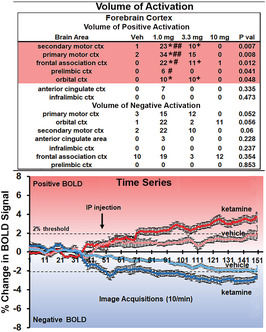

**Abbreviations**
BOLD: blood oxygen levle dependent
FOV: field of view
HASTE: Half Fourier Acquisition Single Shot Turbo Spin Echo
NMDAR: N‐methyl‐d‐aspartate receptormethyl‐d‐aspartate receptor
pHMRI: pharmacological magnetic resonance imaging
RARE: rapid acquisition with relaxation enhancement
ROI: region of interest
rsFC: resting state functional connectivity
TE: time to echo
TRD: treatment‐resistant depression

## 1 INTRODUCTION

One of the more recent epidemiological studies on adult major depression in the United States, reports lifetime prevalence of over 20%.^1^ About 7% of Americans aged 18 and older, around 16 million individuals, are affected by depression every year. The economic burden is staggering, with an estimated cost of $210 billion per year as of 2010.^2^ Psychotherapy is currently the first‐line treatment and is often accompanied by prescribed antidepressants such as monoamine oxidase inhibitors or selective serotonin reuptake inhibitors.^3^ If no improvement in symptoms is observed within 4 weeks, other drugs or a combination of drugs are utilized. Patients that do not respond to existing standard treatments are diagnosed with treatment‐resistant depression (TRD) requiring more intense and/or invasive therapy such as electroconvulsive shock therapy, vagus nerve stimulation, or deep brain stimulation.^4^ However, there is accumulating evidence that the administration of (±) ketamine or its S‐isomer esketamine is highly efficacious for treating TRD.^5^ Yet, ketamine's use is controversial and is currently only used as a last‐line treatment.^6^ Ketamine stands out because the therapeutic effects are often seen within 24 h of IV administration, making it an attractive form of therapy. However, ketamine's mechanism of action on the brain that leads to the alleviation of depression is not fully elucidated, although numerous studies in humans and animals have found that the prefrontal cortex and hippocampus are affected by the drug.^7^, ^8^, ^9^, ^10^, ^11^, ^12^, ^13^

Current theories on the pathology of depression focus on abnormalities or dysfunction of emotional circuits in the brain. Neuroimaging of patients with depression has led to several models proposing dysfunction of specific brain regions that result in depression. Dysregulation in mesolimbic circuitry is thought to play a key role in anhedonia and motivational processing in depression.^14^ Hypoactivity in frontal cortical regions involving emotional processing are thought to significantly contribute to depression,^15^ in addition to dysfunction in the regulation of striatal and subcortical regions by the prefrontal cortex.^16^

Racemic ketamine is a promiscuous molecule, noted as an N‐methyl‐d‐aspartate receptor (NMDAR) antagonist, but with a significant affinity for serotonin, dopamine, cholinergic, and opioid receptors.^17^ (±) Ketamine is active across a broad range of doses with diverse behavioral effects in animals from enhanced locomotor activity at the lower range (1 mg/kg),^18^ to antidepression, analgesic, psychomimetic and anesthetic effects at the higher range (30 mg/kg).^19^ While there have been several imaging studies on racemic ketamine in awake and anesthetized animals that focus on brain activity associated with the higher dose range, this study was undertaken to look at the low dose effects of esketamine the more active isomer of (±) ketamine recently approved by the US Food and Drug Administration for treating TRD.^20^ The rapid and widespread effects of esketamine were tested using blood oxygen level‐dependent (BOLD) pharmacological magnetic resonance imaging (phMRI) for the first time on awake mice. Dose‐dependent changes were noted in the forebrain, including the prefrontal cortex and hippocampus with only modest changes in reward circuitry.

## 2 METHODS

### 2.1 Animal usage

Male C57BL/J6 mice (*n* = 32) approximately 100 days of age and weighing between 28 and 30 gm were obtained from Charles River Laboratories (Wilmington, Massachusetts, USA). Mice were maintained on a 12:12 h light–dark cycle with lights on at 07:00 h and allowed access to food and water ad libitum. All mice were acquired and cared for in accordance with the guidelines published in the Guide for the Care and Use of Laboratory Animals (National Institutes of Health Publications No. 85–23, Revised 1985) and adhered to the National Institutes of Health and the American Association for Laboratory Animal Science guidelines. The protocols used in this study complied with the regulations of the Institutional Animal Care and Use Committee at the Northeastern University and adhere to the ARRIVE guidelines for reporting in vivo experiments in animal research.^21^

### 2.2 Drug preparation and administration

Esketamine was purchased from Sigma Chemical and dissolved in 0.9% NaCl for I.P. injections. Mice were randomly assigned to one of four groups corresponding to saline vehicle, 1.0, 3.3, or 10 mg/kg esketamine. To deliver drug remotely during the imaging session, a poly‐ethylene tube (PE‐20), approximately 30 cm in length, was positioned in the peritoneal cavity. The range of doses were taken from the literature.^18^, ^22^

### 2.3 Awake mouse imaging

#### 2.3.1 Imaging system

**FIGURE 1:**
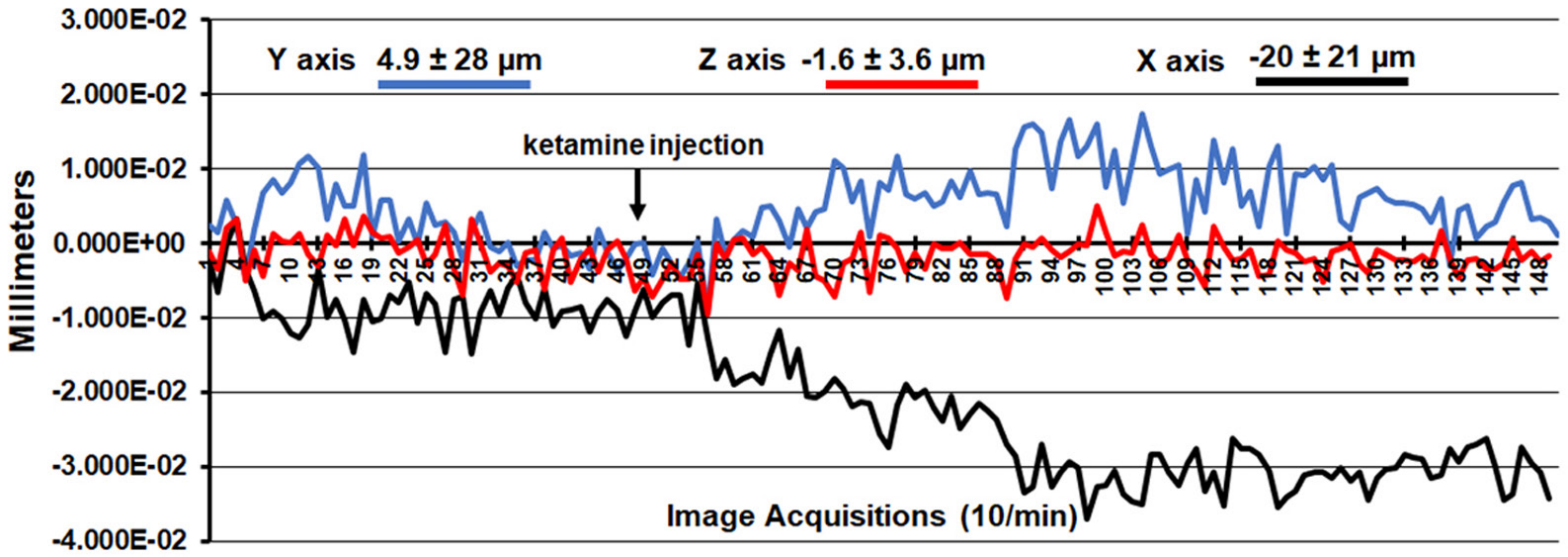
Motion artifact. Shown is the degree of motion artifact recorded over the 15 min imaging protocol. The data are reported as the mean and standard deviation in micrometers for axis Y, Z, and X for the eight mice injected with the 10 mg dose of esketamine. The largest movement in the X axis (left to right head motion) was ca 40 μm for any mouse.

A detailed description of the awake mouse imaging system is published elsewhere.^23^ Notably, we used a quadrature transmit/receive volume coil (ID = 38 mm) that provided both high anatomical resolution and high signal‐to‐noise ratio for voxel‐based BOLD fMRI. Furthermore, the unique design of the mouse holder (Ekam Imaging) fully stabilized the head in a cushioned helmet, minimizing discomfort caused by ear bars and other restraint systems that are commonly used to immobilize the head for awake animal imaging. A movie showing the set‐up of a mouse for awake imaging is available at http://www.youtube.com/watch?v =W5Jup13isqw. The effectiveness of this passive restraining system can be judged by the minimal level of motion artifact recorded over the 15 min imaging session with the injection of 10 mg/kg dose of esketamine as shown in Figure 1. Indeed, the average displacement in any orthogonal direction over the entire 15 min scanning session did not exceed 20 μm.

#### 2.3.2 Acclimation

A week prior to the first imaging session, all mice were acclimated to the head restraint and to the noise of the scanner. Mice were first secured in the holding system under 1–2% isoflurane anesthesia. Upon regaining consciousness, mice were placed for up to 30 min in a “mock MRI scanner” environment, consisting of an enclosed black box equipped with an audio recording of MRI pulses. The acclimation protocol was repeated over four consecutive days to reduce autonomic nervous system‐induced effects during awake animal imaging (e.g., changes in heart rate, respiration, corticosteroid levels, and motor movements), with the goal of improving contrast‐to‐noise ratios and image quality.^24^ Still, other labs have focused on longer periods of acclimation to minimize stress during awake imaging^25^, ^26^ Following acclimation mice were randomized into four groups with eight mice per group.

#### 2.3.3 BOLD phMRI image acquisition and pulse sequence

**FIGURE 2:**
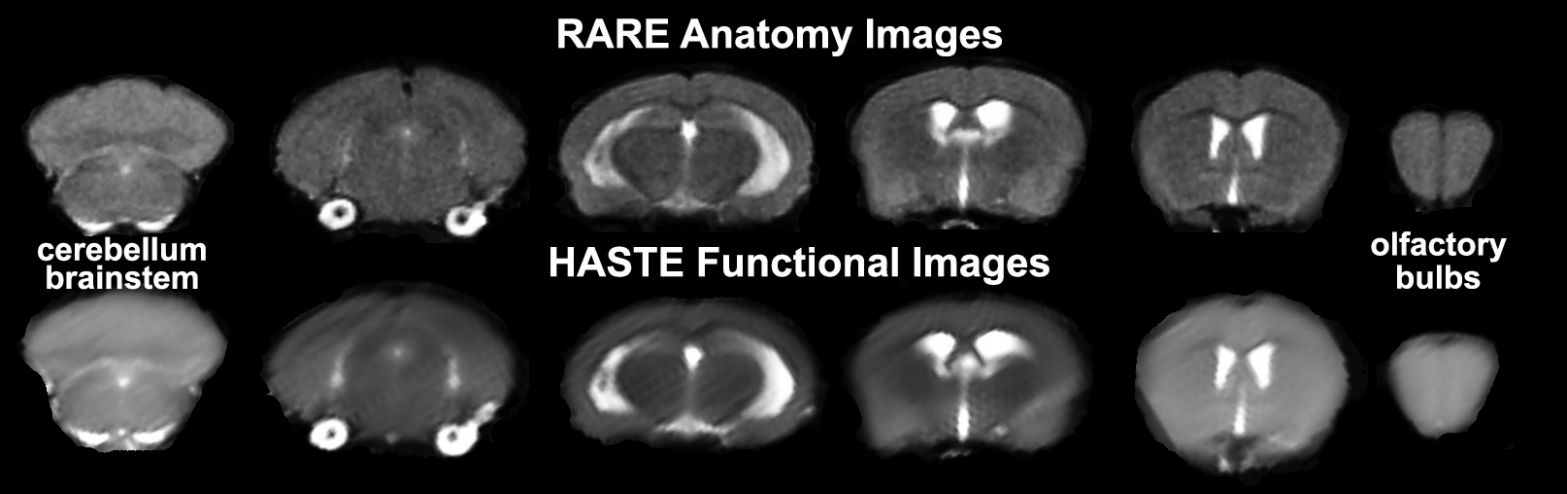
Neuroanatomical fidelity. Shown are representative examples of brain images collected during a single imaging session using a multi‐slice spin echo, RARE (rapid acquisition with relaxation enhancement) pulse sequence. The row on the top shows axial sections collected during the anatomical scan taken at the beginning of each imaging session using a data matrix of 256 × 256, 18 slices in a field of view of 3.0 cm. The row below shows the same images but collected for functional analysis using HASTE, a RARE pulse sequence modified for faster acquisition time. These images were acquired using the same field of view and slice anatomy but a larger data matrix of 96 × 96. Note the anatomical fidelity between the functional images and their original anatomical image. The absence of any distortion is necessary when registering the data to the atlas to resolve 134 segmented brain areas.

Experiments were conducted using a Bruker Biospec 7.0 T/20‐cm USR horizontal magnet (Bruker) and a 20‐G/cm magnetic field gradient insert (ID = 12 cm) capable of a 120‐μsec rise time. At the beginning of each imaging session, a high‐resolution anatomical data set was collected using the RARE pulse sequence (18 slices; 0.75 mm; FOV 1.8 cm^2^; data matrix 128 × 128; TR 2.1 s; TE 12.4 ms; Effect TE 48 ms; NEX 6; 6.5 min acquisition time). Functional images were acquired using a multi‐slice Half Fourier Acquisition Single Shot Turbo Spin Echo (HASTE) pulse sequence (18 slices; 0.75 mm; FOV 1.8 cm; data matrix 96 × 96; TR 6 s; TE 4 ms; Effective TE 24 ms; 15 min acquisition time; in‐plane resolution 187.5 μm^2^). This spatial resolution is enough to delineate the bilateral habenula (ca 4–5 voxels for each side) but not between lateral and medial habenula. The use of spin echo to acquire the functional images was necessary to achieve the high anatomical fidelity required for data registration to the mouse MRI atlas as shown in Figure 2. Each functional imaging session consisted of uninterrupted data acquisitions (whole brain scans) of 150 scan repetitions or acquisitions for a total elapsed time of 15 min. The control window included the first 50 scan acquisitions (18 slices acquired in each), covering a 5 min baseline. Following the control window, an I.P. injection of vehicle or esketamine was given followed by another 100 acquisitions over a 10 min period. Masaki et al., reported brain levels of esketamine (10 mg/kg) given I.P. peaked within 5 min of injection accompanied by an increase in locomotor behavior.^12^ The order of drug doses was randomized over the scanning sessions. One of the mice in the vehicle group and two of mice in the group receiving the 3.3 mg/kg dose of esketamine were eliminated because of excessive motion artifact (movement greater than the dimensions of half of one voxel, ca 90 μm in plane).

#### 2.3.4 Imaging data analysis

The dose‐dependent effect of esketamine on brain activity was quantified through positive and negative percent changes in BOLD signal relative to baseline. The statistical significance of these changes was assessed for each voxel (~15 000 per subject, in their original reference system) with independent Student's *t*‐tests, with a 2% threshold to account for normal fluctuations of BOLD signal in the awake rodent brain.^27^ As a result of the multiple t‐tests performed, a false‐positive detection‐controlling mechanism was introduced,^28^ to ensure that, on average, the false‐positive detection rate remained below 0.05. The following formula was used:

(3) $$P_{i}\leq \frac{i}{V}\frac{q}{c\left(V\right)},$$

where $P_{i}$ is the *p‐*value from the t‐test at the *i‐*th pixel within the region of interest (ROI) containing V pixels, each ranked based on its probability value. The false‐positive filter value q was set to 0.2 for our analysis, and the predetermined constant $c\left(V\right)$ was set to unity,^29^ providing conservative estimates for significance. Pixels that were statistically significant retained their relative percentage change values, while all other pixel values were set to zero. A 95% confidence level, two‐tailed distributions, and heteroscedastic variance assumptions were employed for the t‐tests.

Voxel‐based percent changes in BOLD signal were combined across subjects within the same group to build representative functional maps. To this end, all images were first aligned and registered to a 3D Mouse Brain Atlas© with 134 segmented and annotated brain regions (Ekam Solutions). The co‐registrational code SPM8 was used with the following parameters: Quality: 0.97, Smoothing: 0.35 mm, Separation: 0.50 mm. Gaussian smoothing was performed with an FWHM of 0.8 mm. Image registration involved translation, rotation, and scaling, independently and in all three dimensions. All applied spatial transformations were compiled into a matrix $\left[T_{j}\right]$ for the j‐th subject. Every transformed anatomical pixel location was tagged with a brain area to generate fully segmented representations of individual subjects within the atlas.

Next, composite maps of the percent changes in BOLD signal were built for each experimental group. Each composite pixel location (row, column, and slice) was mapped to a voxel of the j‐th subject by virtue of the inverse transformation matrix ${\left[T_{j}\right]}^{-1}$. A tri‐linear interpolation of subject‐specific voxel values determined their contribution to the composite representation. The use of the inverse matrices ensured that the full composite volume was populated with subject inputs. The average of all contributions was assigned as the percent change in BOLD signal at each voxel within the composite representation of the brain for the respective experimental group. The number of activated voxels in each of the 134 regions was then compared between the control and esketamine doses using a Kruskal–Wallis test statistic. Post hoc analyses were performed with a Wilcoxon rank‐sum test. Brain regions were considered statistically different between experimental groups when *p* ≤ .05.

## 3 RESULTS

Shown in Table 1 is a truncated list of 54 out of 134 brain areas ranked in order of their significance for change in positive BOLD volume of activation (number of voxels). Reported is the median number of voxels significantly activated 10 min post I.P. injection of vehicle (Veh), 1, 3.3, and 10 mg/kg of esketamine with a critical value α < .05 and the omega square (ω Sq) for effect size. Using a false detection rate for multi‐comparisons gives a significance level of *p* ≤ .08. Table S1 of positive BOLD volume of activation is provided for all 134 brain areas. The 1.0 mg/kg dose was most effective in increasing BOLD signal. There are no brain areas that showed a dose‐dependent increase in brain activity. Instead, there were numerous brain areas that showed a dose‐dependent decrease in brain activity with esketamine, for example, somatosensory cortex, motor cortex, frontal association cortex, caudate/putamen, and CA1, CA3, hippocampus. Other areas like the paramedian lobule, gigantocellularis reticularis, cerebral peduncle, anterior hypothalamus, and subiculum showed a U‐shaped dose‐response with 3.3 mg/kg having the smallest effect on BOLD signal. Similarly, Table 2 shows a list of 52 brain areas ranked in order of their significance for change in negative BOLD volume of activation. The false detection rate for multi‐comparisons gives a significance level of *p* ≤ .077. A Table S2 for negative BOLD volume of activation is provided for all 134 brain areas. There was no clear descending or ascending dose–response in negative BOLD. In 27/52 brain areas 1 mg had the greatest effect while in 15/52 areas 3.3 mg was the most effective. Areas like the basal amygdala and gigantocellularis reticularis presented with greater negative BOLD to the 10 mg dose, while areas like the flocculus of the cerebellum, olfactory tubercles, and medullary reticular area were all comparable in their response across doses.

**TABLE 1:** Change in postive BOLD volume of activation with esketamine

Positive volume of activation						
Brain area	Veh	1.0 mg	3.3 mg	10 mg	*p* value	ω Sq
Auditory ctx	0	20	39	4	.002	0.483
Lateral lemniscus	0	14	0	9	.002	0.467
Primary somatosensory ctx	12	164	136	8	.003	0.434
Paramedian lobule	1	16	4	37	.003	0.420
Pontine area	0	32	2	37	.003	0.416
Gigantocellaris reticular area	0	26	0	39	.003	0.416
Entorhinal ctx	27	151	75	115	.004	0.410
Cerebral peduncle	0	46	8	19	.005	0.393
Lateral septal area	0	18	4	0	.005	0.392
Secondary somatosensory ctx	4	45	27	0	.005	0.386
Secondary motor ctx	1	23	10	0	.007	0.362
Habenular area	0	3	0	0	.007	0.354
Primary motor ctx	2	34	15	0	.008	0.342
Lateral dorsal thalamic area	0	6	1	0	.008	0.342
Ventral medial hypothalamic area	0	5	0	8	.009	0.337
7th cerebellar lobule	0	1	0	9	.009	0.337
Subiculum	0	16	9	32	.009	0.335
Principal sensory n. trigeminal	0	25	5	26	.009	0.333
Glomerular layer	0	26	6	7	.01	0.328
Prepositus area	0	1	0	0	.011	0.314
Frontal association ctx	0	22	11	1	.012	0.308
Lateral posterior thalamic area	0	1	0	0	.014	0.299
Parvicellular reticular area	0	11	1	18	.015	0.293
Medial dorsal thalamic area	0	4	0	0	.015	0.289
Posterior thalamic area	0	4	0	0	.016	0.284
Parafascicular thalamic area	0	0	0	0	.018	0.278
Anterior thalamic area	0	11	2	0	.018	0.277
Central amygdaloid area	0	3	7	0	.019	0.271
Central medial thalamic area	0	1	0	0	.02	0.265
Flocculus cerebellum	6	46	44	52	.021	0.261
Fimbria hippocampus	4	18	9	0	.022	0.259
Ventral pallidum	0	6	0	1	.022	0.257
Accumbens shell	0	2	0	0	.024	0.250
Lateral geniculate	0	0	3	0	.026	0.245
Tenia tecta ctx	2	14	0	5	.026	0.243
Caudate putamen	12	93	45	0	.026	0.243
Ventral thalamic area	0	40	26	4	.028	0.237
Spinal trigeminal nuclear area	8	52	12	35	.029	0.234
Anterior hypothalamic area	0	19	1	16	.031	0.230
Intermediate reticular area	1	9	1	12	.031	0.229
Lateral rostral hypothalamic area	0	8	5	4	.032	0.227
CA3	2	36	12	3	.032	0.226
Insular rostral ctx	21	38	50	4	.036	0.215
Dorsal medial hypothalamic area	0	4	0	2	.04	0.208
Lateral amygdaloid area	0	0	2	0	.04	0.207
Prelimbic ctx	0	6	0	0	.041	0.038
Basal amygdaloid area	0	22	3	9	.041	0.204
Pituitary	1	9	0	7	.043	0.201
Medial preoptic area	0	12	0	4	.044	0.198
CA1	2	57	31	5	.046	0.194
Pontine reticular nucleus caudal	3	16	3	25	.047	0.193
Facial nucleus	0	3	0	4	.048	0.191
Orbital ctx	0	10	10	0	.048	0.191
Pontine reticular nucleus oral	1	20	2	11	.048	0.190

**TABLE 2:** Negative BOLD volume of activation

Negative volume of activation						
Brain area	Veh	1.0 mg	3.3 mg	10 mg	*p* value	ω Sq
Medial geniculate	0	11	0	0	0	0.524
Zona incerta	0	6	0	0	.001	0.475
Habenular area	0	2	4	0	.001	0.498
Caudal piriform ctx	5	61	31	37	.002	0.409
Anterior pretectal thalamic area	0	6	3	0	.002	0.434
Insular caudal ctx	0	24	13	2	.002	0.409
Caudate putamen	0	86	39	48	.002	0.427
External capsule	0	3	1	4	.003	0.397
Medullary reticular ventral area	1	17	23	21	.004	0.392
Second cerebellar lobule	0	11	17	1	.005	0.351
Third cerebellar lobule	0	5	1	0	.006	0.343
Bed n. stria terminalis	0	4	3	0	.006	0.366
Medial dorsal thalamic area	0	5	6	0	.006	0.378
Tenth cerebellar lobule	0	2	4	0	.006	0.366
Corpus callosum	0	14	5	3	.008	0.321
Anterior thalamic area	0	6	16	0	.008	0.347
Anterior hypothalamic area	0	10	25	13	.009	0.328
Lateral caudal hypothalamic area	0	3	0	3	.01	0.269
Superior colliculus	0	29	7	2	.01	0.291
Principal sensory n. trigeminal	0	14	8	3	.011	0.313
Cerebral peduncle	0	12	17	2	.012	0.316
Auditory ctx	0	12	10	1	.012	0.325
Lateral rostral hypothalamic area	0	6	0	2	.013	0.288
Entorhinal ctx	11	80	85	44	.013	0.316
Lateral septal area	0	20	17	10	.014	0.314
Basal amygdaloid area	0	10	4	27	.015	0.286
Rostral piriform ctx	5	69	57	32	.015	0.274
Lateral paragigantocellular area	0	8	14	13	.015	0.260
Central amygdaloid area	0	6	0	4	.015	0.287
Medial preoptic area	0	3	14	11	.016	0.278
Cortical amygdaloid area	5	41	37	31	.016	0.270
Central medial thalamic area	0	0	0	0	.018	0.212
Inferior colliculus	0	29	1	0	.018	0.215
Paraventricular thalamic area	0	0	7	0	.019	0.281
Endopiriform area	0	7	0	0	.019	0.246
Reticular thalamic area	0	5	1	0	.022	0.246
CA3	2	33	22	4	.022	0.254
CA1	1	42	37	5	.025	0.241
Cerebellar nuclear area	0	2	4	1	.025	0.258
Flocculus cerebellum	5	23	22	22	.025	0.248
Olivary complex	0	8	0	8	.027	0.200
Fifth cerebellar lobule	0	10	9	0	.028	0.231
Olfactory tubercles	0	7	13	11	.028	0.228
Pontine reticular nucleus oral	0	11	1	2	.029	0.220
Posterior hypothalamic area	0	0	0	2	.031	0.137
Vestibular area	0	11	18	0	.032	0.225
Anterior commissure	0	1	0	0	.038	0.231
Periaqueductal gray	0	35	11	0	.041	0.165
Accumbens core	0	11	1	0	.042	0.238
Lateral geniculate	0	1	1	0	.044	0.224
Gigantocellaris reticular area	3	11	24	27	.046	0.221
Dorsal raphe	0	0	2	3	.048	0.191

**FIGURE 3:**
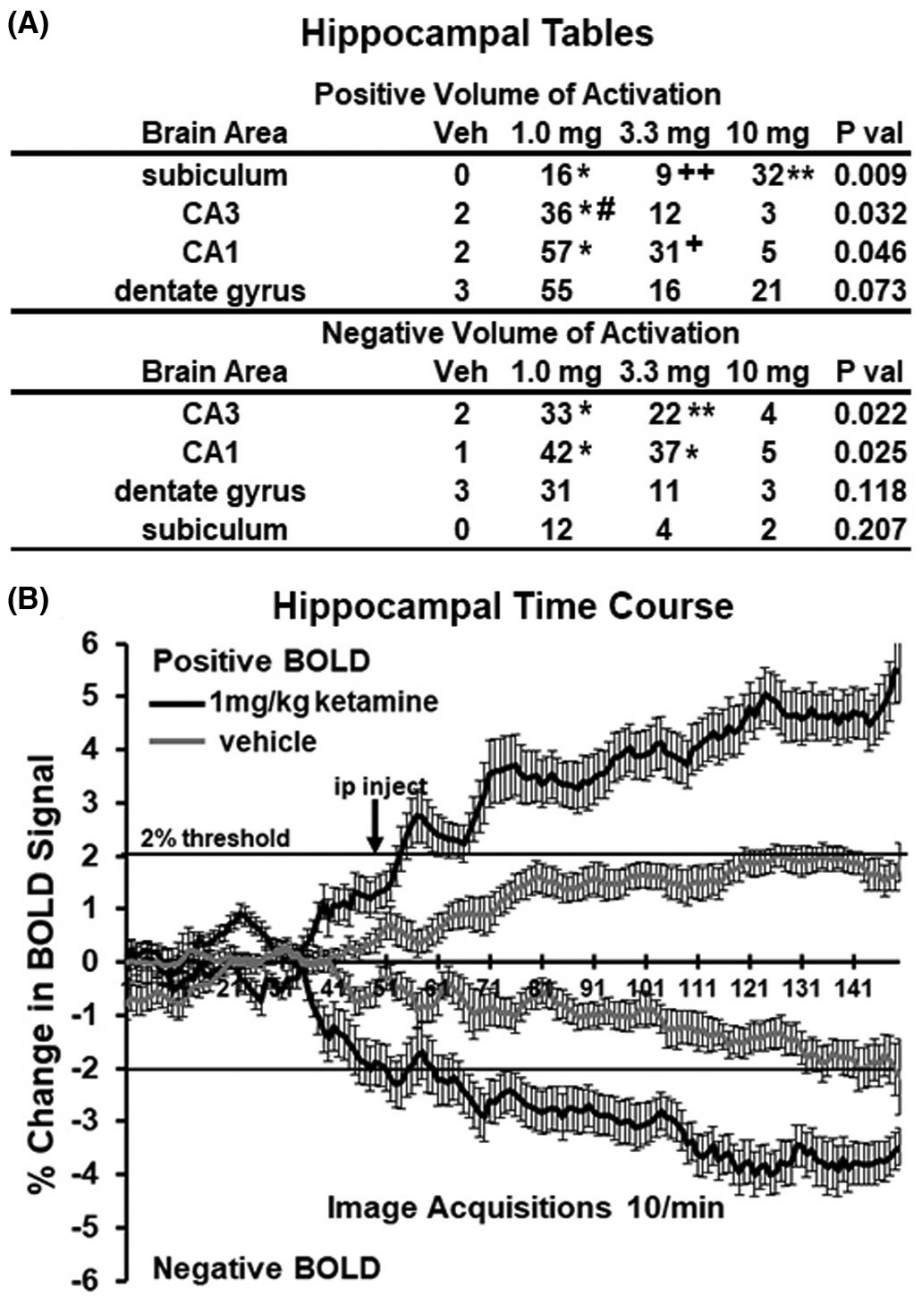
BOLD signal changes in the hippocampus. The tables (A) show the positive and negative changes in BOLD volume of activation (voxel numbers) for each of the doses of esketamine in the hippocampus. Positive volume of activation: (*p < 0.05, 1.0 mg vs Veh); (#*p* < 0.05, 1.0 mg CA3 vs 10 mg CA3); (+*p* < 0.05, 3.3 mg CA1 vs 10 mg CA1); (++*p* < 0.01, 3.3 mg subiculum vs 10 mg subiculum). Negative volume of activation: (*p < 0.05, 1.0 mg vs Veh, 3.3 mg CA1 vs Veh); (**p < 0.01, 3.3 mg CA3 vs Veh). The time series below (B) show positive and negative changes in BOLD signal over the 15 min imaging period comparing the 1.0 mg dose of esketamine to vehicle. For positive change in signal, esketamine was greater than vehicle (*F*_(1,58)_ = 27.40, *p* < 0.0001;). For negative change in signal, esketamine was greater than vehicle (*F*_(1,58)_ = 23.39, *p* < 0.0001).

**FIGURE 4:**
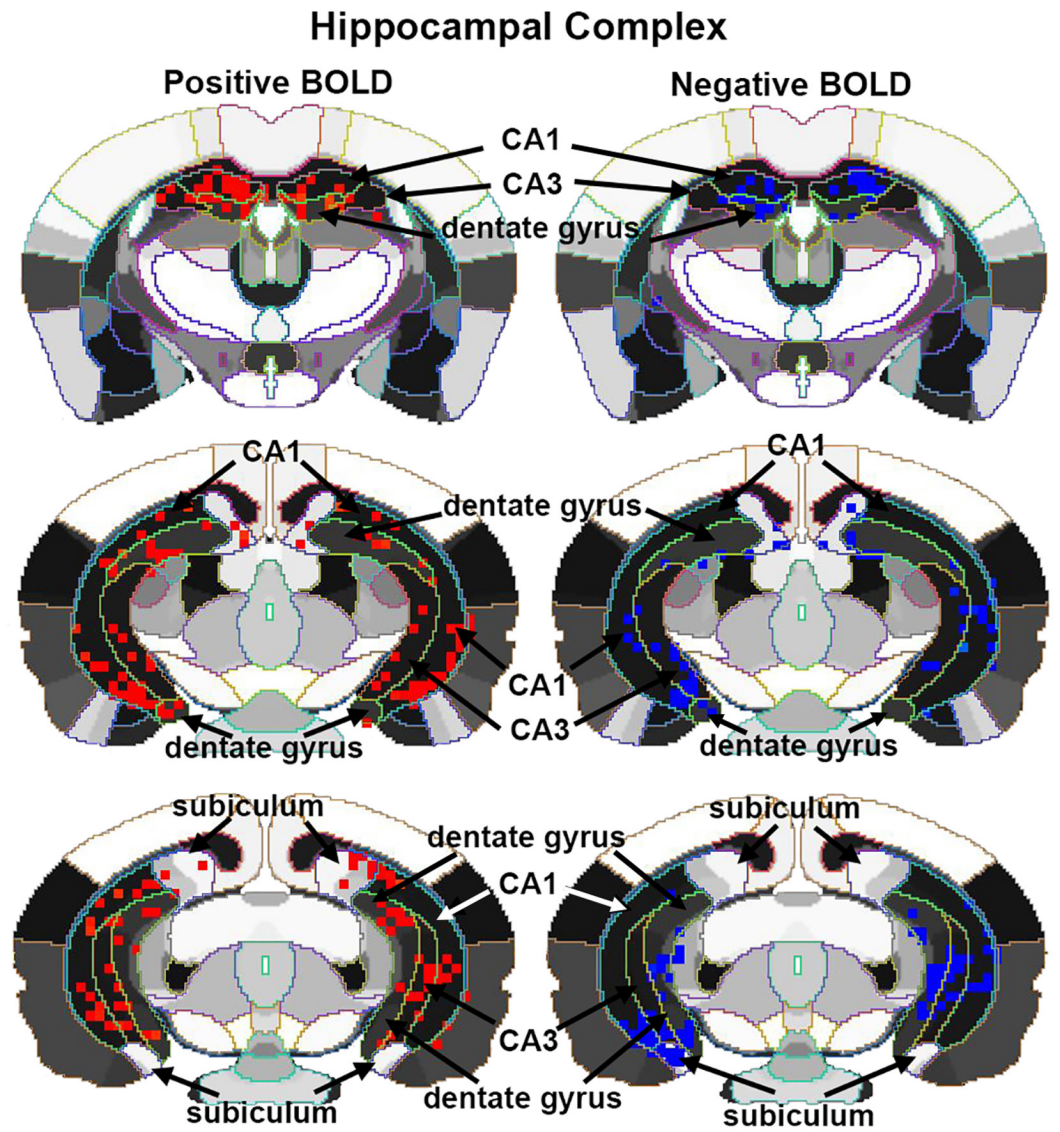
Localization of negative and positive BOLD voxels. The 2D maps show the localization of positive and negative BOLD voxels collected during the scanning session registered to the segmented MRI mouse atlas. These composites represent the average number of voxels from the eight mice injected with 1.0 mg/kg of esketamine. Sections are aligned rostral (top) to caudal (bottom).

Shown in Figure 3 are volume of activation (A) and time series (B) for changes in positive and negative BOLD in the hippocampus following esketamine injection. Three of the four areas comprising the hippocampal complex, for example, CA1, CA3, and dentate gyrus, show a significantly greater volume of activation (voxel numbers) for 1.0 mg/kg as compared to vehicle (*p < .05). The 1 mg dose for CA3 was also greater than the 10 mg/kg dose (#*p* < .05). The 3.3 mg/kg dose was greater than the 10 mg dose for CA1 (+*p* < .05) but less for the subiculum (++*p* < .01). The change in negative BOLD for CA3 and CA1 was significantly affected by the 1.0 and 3.3 mg/kg doses of esketamine (*p < .05; **p < .01). The time series shows percent change in BOLD over 10 min following injection of 1.0 mg/kg esketamine. The difference in positive BOLD signal over time between vehicle and esketamine was statistically significant (2‐way ANOVA, *F*_(1,58)_ = 27.40, *p* < .0001; esketamine > Veh). The difference in negative BOLD between vehicle and esketamine was also significant (*F*_(1,58)_ = 23.39, *p* < .0001; esketamine > Veh). Vehicle for negative and positive BOLD never exceeded the 2% threshold set for awake imaging. The location of the simultaneously affected positive and negative BOLD voxels in the hippocampal complex are shown in the 2D maps in Figure 4. The precise location of the positive and negative voxels is shown registered to the MRI mouse atlas. These composites are the average number of voxels showing a significant increase or decrease above or below baseline for 1.0 mg/kg esketamine (*n* = 8).

**FIGURE 5:**
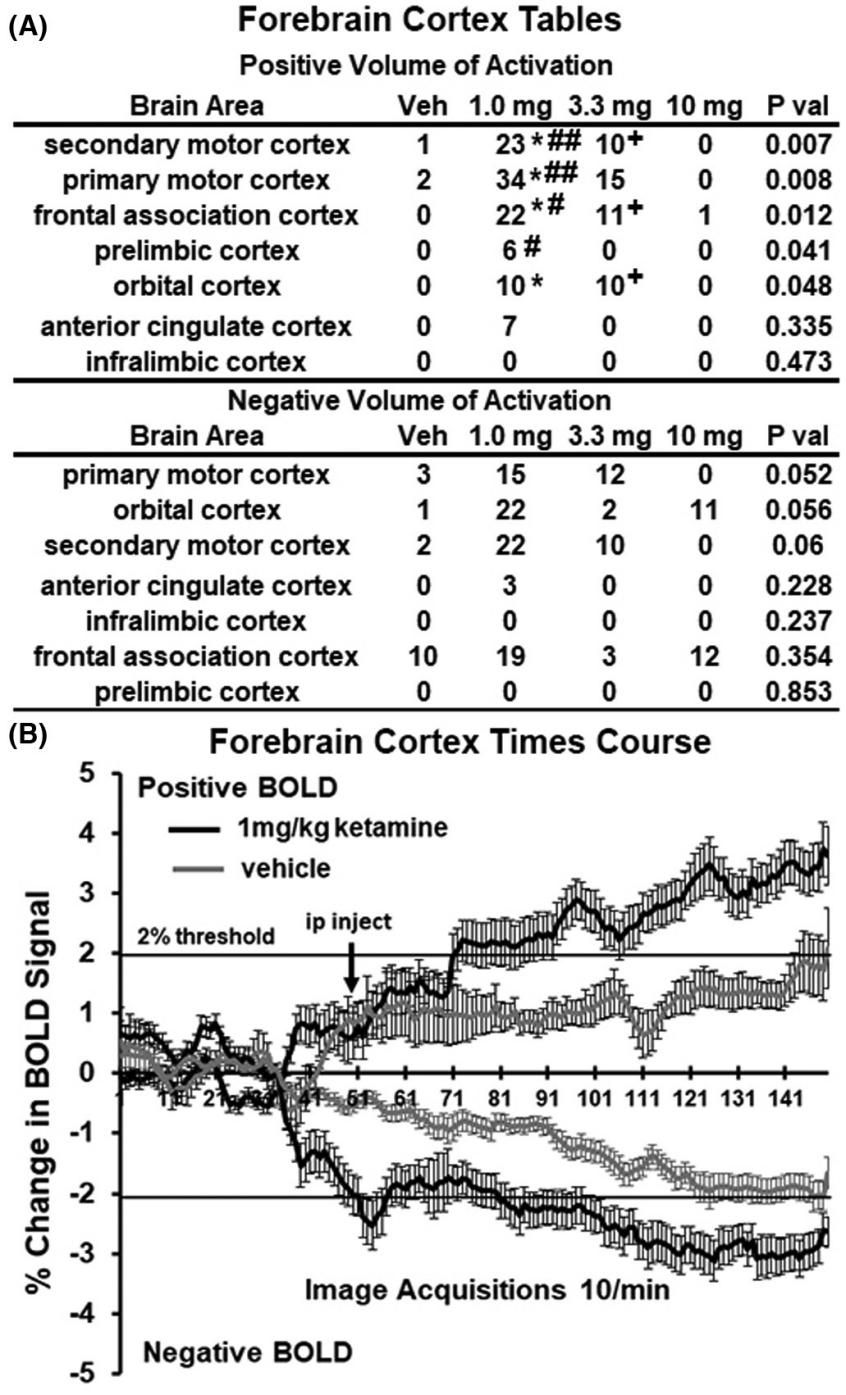
BOLD signal changes in the forebrain. The tables (A) show the positive and negative changes in BOLD volume of activation (voxel numbers) for each of the doses of esketamine in areas of the forebrain. Denoted areas are significantly different from vehicle or from each other. (*p < 0.05, 1.0 mg vs vehicle); (#*p* < 0.05 and ##*p* < 0.01, 1.0 mg vs 10 mg); (+*p* < 0.05, 3.3 mg vs 10 mg). The time series below (B) show positive and negative changes in BOLD signal over the 15 min imaging period comparing the 1.0 mg dose of esketamine to vehicle. For positive change in signal, esketamine was greater than vehicle (*F*_(1,103)_ = 8.074, *p* < 0.0054). For negative change in signal, esketamine was greater than vehicle (*F*_(1,103)_ = 17.58, *p* < 0.0001).

Figure 5 shows the volume of activation (A) and time series (B) for changes in positive and negative BOLD in the forebrain following esketamine injection. Four of the seven areas comprising the forebrain show a significant increase in BOLD over vehicle with 1.0 mg (*p < .05). The 1.0 mg/kg dose was also significantly greater than the 10 mg/kg dose (#*p* < .05, ##*p* < .01). The 3.3 mg/kg dose was also significantly greater than the 10 mg/kg dose of esketamine (+*p* < .05). No brain areas in the forebrain showed a significant negative BOLD volume of activity with esketamine. The difference in positive BOLD signal over time between vehicle and esketamine was statistically significant (2‐way ANOVA, *F*_(1,103)_ = 8.074, *p* < .0054; esketamine > Veh). The difference in negative BOLD between vehicle and esketamine was also significant (*F*_(1,103)_ = 17.58, *p* < .0001; esketamine > Veh). Vehicle for negative and positive BOLD never exceeded the 2% threshold set for awake imaging.

**FIGURE 6:**
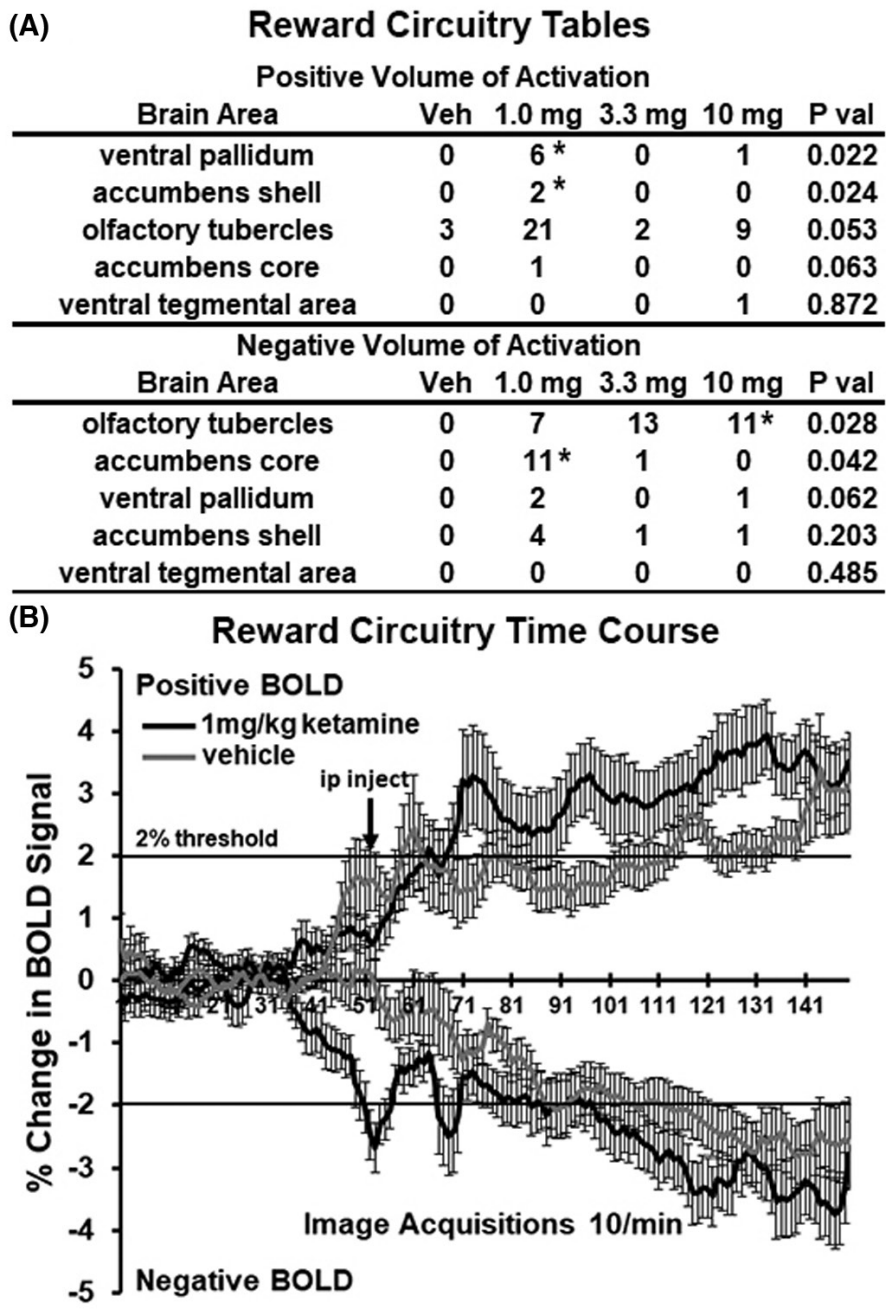
Bold signal changes in reward areas. The tables (A) show the positive and negative changes in BOLD volume of activation (voxel numbers) for each of the doses of esketamine in the areas of reward circuitry. Denoted areas are significantly different from vehicle. (*p < 0.05, 1.0 mg vs Veh, 10 mg vs Veh). There was no significant difference in positive or negative BOLD signal over time (B) between vehicle and esketamine (2‐way ANOVA, *F*_(1,73)_ = 1.431, *p* = .2355; *F*_(1,73)_ = 3.321, *p* = .0725, respectively).

Shown in Figure 6 are the volume of activation (A) and time series (B) for changes in positive and negative BOLD in the reward circuitry following esketamine injection. The areas of the ventral pallidum and accumbens shell showed a significant increase in BOLD to 1.0 mg versus veh (*p < .05). There was no significant difference in positive or negative BOLD signal over time between vehicle and esketamine (two‐way ANOVA, *F*_(1,73)_ = 1.431, *p* = .2355; *F*_(1,73)_ = 3.321, *p* = .0725, respectively).

## 4 DISCUSSION

As noted, racemic ketamine is a promiscuous molecule, with an affinity for multiple receptors in addition to NMDAR.^17^ Ketamine is active across a broad range of doses with diverse behavioral effects, from enhanced locomotor activity at low doses to anesthesia at higher doses. While there have been several imaging studies on racemic ketamine in awake and anesthetized animals that focus on brain activity associated with higher doses, these studies were undertaken to look at the effect of esketamine, the more active isomer of ketamine approved for the treatment of TRD, at low doses ranging from 1.0 to 10 mg/kg. These doses spanning a magnitude in difference have been used in several rodent studies for racemic ketamine.^13^, ^30^, ^31^, ^32^, ^33^ To our surprise, the lowest dose of esketamine had the greatest effect on positive BOLD activity. A majority of brain areas showed a decreasing BOLD dose–response. The areas of the brain most sensitive to the 1.0 mg/kg dose were the prefrontal cortex and hippocampus. These data are discussed in the context of previous imaging studies and the neural circuits associated with psychosis and depression.

Tang et al., tested ketamine in awake rats at a dose of 3 mg/kg^13^ and showed a peak response within the first 15 min highlighted by positive BOLD activation in prefrontal cortex, striatum, thalamus, and hippocampus. Chin and colleagues working with awake rats reported a 30 mg/kg dose of ketamine caused significant increases in BOLD signal in the prefrontal cortex, anterior cingulate, and hippocampus while decreasing BOLD in the ventral tegmental areas, substantia nigra, and periaqueductal gray.^34^ Awake rats treated with 10 mg/kg of esketamine show an increase in prefrontal cortex and anterior cingulate using contrast enhanced, cerebral blood flow imaging.^12^ Despite the use of anesthesia, Littlewood et al., reported a comparable increase in BOLD signal in rats in the orbital cortex and hippocampus following 25 mg/kg of racemic ketamine and esketamine.^35^ Anesthetized mice given 30 mg/kg ketamine show increased resting state BOLD functional connectivity (rsFC) between hippocampus and prefrontal cortex in females but not males.^19^ In the first study to look at the dose‐dependent effect of racemic ketamine on rsFC, Gass and colleagues challenged anesthetized rats with 5, 10, and 25 mg/kg doses of ketamine during the scanning session and reported a dose‐dependent increase in coupling within and between the prefrontal cortex and hippocampus.^10^ In a subsequent, study these researchers showed the effect of ketamine given to healthy volunteers (0.5 mg/kg) or anesthetized rats (25 mg/kg) induced a robust increase in coupling between the prefrontal cortex and hippocampus.^36^ Indeed, there have been many studies in humans looking at subanesthetic effects of ketamine on brain activity. One of the first used PET imaging of regional cerebral blood flow to show a dose‐dependent increase in blood flow to the frontal and cingulate cortices, caudate/putamen, and cerebellum in healthy volunteers.^37^ De Simoni and colleagues reported increases in prefrontal and hippocampal activity in healthy volunteers in a phMRI study using low doses of racemic ketamine (ca 1 mg/kg).^8^ Studies looking at rsFC in healthy volunteers reported changes in coupling in somatosensory networks involved in pain processing with esketamine^38^ and prefrontal cortex networks involved in depression,^39^ psychosis^40^ and working memory^41^ using racemic ketamine.

To the best of our knowledge, this is the first multidose, phMRI BOLD imaging study of esketamine in awake mice. Furthermore this is the only imaging study, awake or anesthetized, to evaluate the efficacy of 1 mg/kg of esketamine, the lowest dose we know of to have a significant effect in a mouse model of depression.^31^ The increase in the motor activity reported with low doses of ketamine^18^, ^33^ did not affect the imaging as measures of motion artifact were very low (see Figure 1). We anticipated a dose‐dependent increase in BOLD signal that was never realized. Instead, many brain areas, for example, forebrain, thalamus, hippocampus, and hypothalamus were only sensitive to the lowest 1 mg/kg dose. Other areas like the sensory motor cortices showed a descending dose response. Hindbrain areas like the pons, medulla, and cerebellum were responsive to the 1 mg and 10 mg doses but not to the 3.3 mg dose (U‐shaped curve). Esketamine had a descending, dose‐dependent, negative BOLD effect, and similar U‐shaped response noted above with the 1 mg and 10 mg doses causing the greatest increase in negative BOLD. Interestingly, the greatest negative BOLD response was localized to brain areas associated with processing olfactory information, for example, the rostral and caudal piriform cortices, endopiriform cortex, entorhinal cortex, olfactory tubercles, and cortical amygdala. Olfaction is critical in rodents and guides much of their behavior.^42^ This presumptive decrease in brain activity with esketamine may account for many of the changes in behavior observed in rodents, for example, exploration, fear, and anxiety.^18^, ^22^, ^43^

As noted, ketamine is a promiscuous drug, affecting numerous signaling systems. What could account for the unique dose‐dependent patterns of BOLD activation where over half of the affected brain areas showed a decrease in positive BOLD activation with higher doses while many others showed the U‐shaped curve? These imaging studies were not designed to interfere with the esketamine response using selective drugs aimed at different signaling pathways. Hence, we cannot assign a mechanism(s) to these dose‐response patterns. The organization of brain areas with specific profiles of activation would suggest a heterogenous but regionalized distribution of putative targets, for example, D2 receptors, 5HT2A, and AMPA. Given the complex pharmacology of ketamine, there is no obvious explanation for the pattern of BOLD signal change based on the location of a single target in the brain.

It is hard to compare our results to other preclinical imaging studies because of the difference in doses, racemic forms, murine species, and protocols, that is, awake versus anesthetized. Nonetheless, there are some similarities and corroborating findings, specifically the activation of the prefrontal cortex and hippocampus.^44^ Our low dose (1 mg/kg) of esketamine activation of prefrontal and hippocampal areas corroborates the findings in healthy human volunteers in a ketamine phMRI study.^8^ These volunteers reported little or no subjective response to the racemic ketamine treatment evidence that low doses can impact brain activity without evoking any cognitive or behavioral response. Dysfunction in connectivity between the prefrontal cortex and hippocampus is noted for its involvement in schizophrenia^45^, ^46^ and depression.^47^, ^48^ Carreno and colleagues reported the antidepressant effect of a 10 mg /kg dose of ketamine requires neural connections between the ventral hippocampus and prefrontal cortex.^7^ There are several environmental and genetic models of depression in rodents that highlight the importance of this prefrontal cortex/hippocampal relationship.^49^, ^50^, ^51^, ^52^ Here, we show that 1.0 mg/kg of esketamine significantly increases BOLD volume of activation in several areas that comprise the prefrontal cortex, for example, prelimbic, orbital, motor, and frontal association cortices. Collectively, these areas show a significant change in BOLD signal over time within mins of esketamine injection. Similarly, the hippocampal complex comprising CA1, CA3, and the subiculum showed significant positive BOLD changes for both volume of activation and percent change over time.

The anhedonia and loss of motivation characteristic of depression are thought to be governed, in part, by dysfunction in dopaminergic signaling.^53^ Ketamine has anti‐anhedonia effects on severely depressed patients.^54^, ^55^ Strerpenich and coworkers reported patients with TDR given ketamine (0.5 mg/kg) showed increased connectivity in reward circuitry, for example, orbitofrontal cortex, ventral striatum, ventral tegmental area, and substantia nigra together with improvement in mood.^14^ In our studies, we did not see any significant increase or decrease in BOLD signal in the midbrain dopaminergic areas, for example, ventral tegmental areas, substantia nigra. However, the caudate/putamen, the efferent target of the substantia nigra showed robust positive and negative changes in BOLD volume of activation to the 1.0 and 3.3 mg doses of esketamine. Esketamine increases the volume of activation in the accumbens shell and ventral pallidum, but not the accumbens core or olfactory tubercles, all efferent targets of the ventral tegmental area. The collection of these areas together with the VTA did not show a significant increase in decrease in BOLD signal over time following esketamine injection.

### 4.1 Limitations

The major limitation of this study was the absence of females. Although there appear to be no sex differences in the response to ketamine's positive therapeutic effects on suicidal ideation^56^ and TRD^57^ there are examples in the preclinical literature in rodents reporting behavioral and neurobiological differences between male and females following racemic ketamine treatment.^19^, ^58^, ^59^, ^60^ The other obvious limitation of this study was the omission an experimental arm that tested mice with genetic or environmental risk for depression. The data presented here were collected from what is considered normal, healthy mice. Would the site‐specific and dose‐dependent effects of esketamine have been different if tested in animal models of psychiatric and neurological disorders? We deliberately ran this study on awake normal mice without the confound of environmental stressors, which clearly alter brain function, molecular biology, and cell structure reported in so many animal models of depression. Indeed, our low‐dose data in awake mice parallel the findings in healthy human volunteers showing activation in prefrontal cortex and hippocampus.^8^ This would appear to be the first step in validating the use of “normal” mice for awake imaging to screen new antidepressants.

## 5 CONCLUSION

The present studies were undertaken to examine the global effects of subanesthetic doses of esketamine on brain areas associated with depression. The rapid and widespread effects of esketamine were tested using phMRI in healthy, awake mice. The anticipated dose‐dependent increase in BOLD was not realized; instead, the lowest dose of 1.0 mg/kg had the greatest effect on brain activity. The prefrontal cortex and hippocampus were significantly activated corroborating previous imaging studies in humans and animals. The unexpected sensitivity to the 1.0 mg/kg dose of esketamine could be explained by imaging in fully awake mice without the confound of anesthesia and/or the enhanced activity of the S‐isomer over (±) ketamine.

## AUTHOR CONTRIBUTIONS

Experimental design and manuscript preparation—PK, CFF; Data generation and analysis—KK, RM, WF, RS, SL.

## ACKNOWLEDGMENTS

None.

## DISCLOSURE

C.F.F. has a financial interest in Animal Imaging Research, the company that makes the radiofrequency electronics and holders for animal imaging. C.F.F. and P.K. have a financial interest in Ekam Imaging a contract research organization that provides MRI data analysis services.

## Supporting information


Table S1.

Table S2.

## Data Availability

All data can be accessed through a link to Mendeley.
